# Supplementary material for: The interactome of intact mitochondria by cross-linking mass spectrometry provides evidence for coexisting respiratory supercomplexes
Source: Mol Cell Proteomics. 2017 Dec 8;17(2):216–32. doi: 10.1074/mcp.RA117.000470 (PMC5795388; doi:10.1074/mcp.RA117.000470)
Supplement: Supplemental Data [file supp_RA117.000470_133922_0_supp_23978_fzffwf.zip › spectra_annotation/mito_spectra_annotation/431-1-2-1-6-1.pdf]

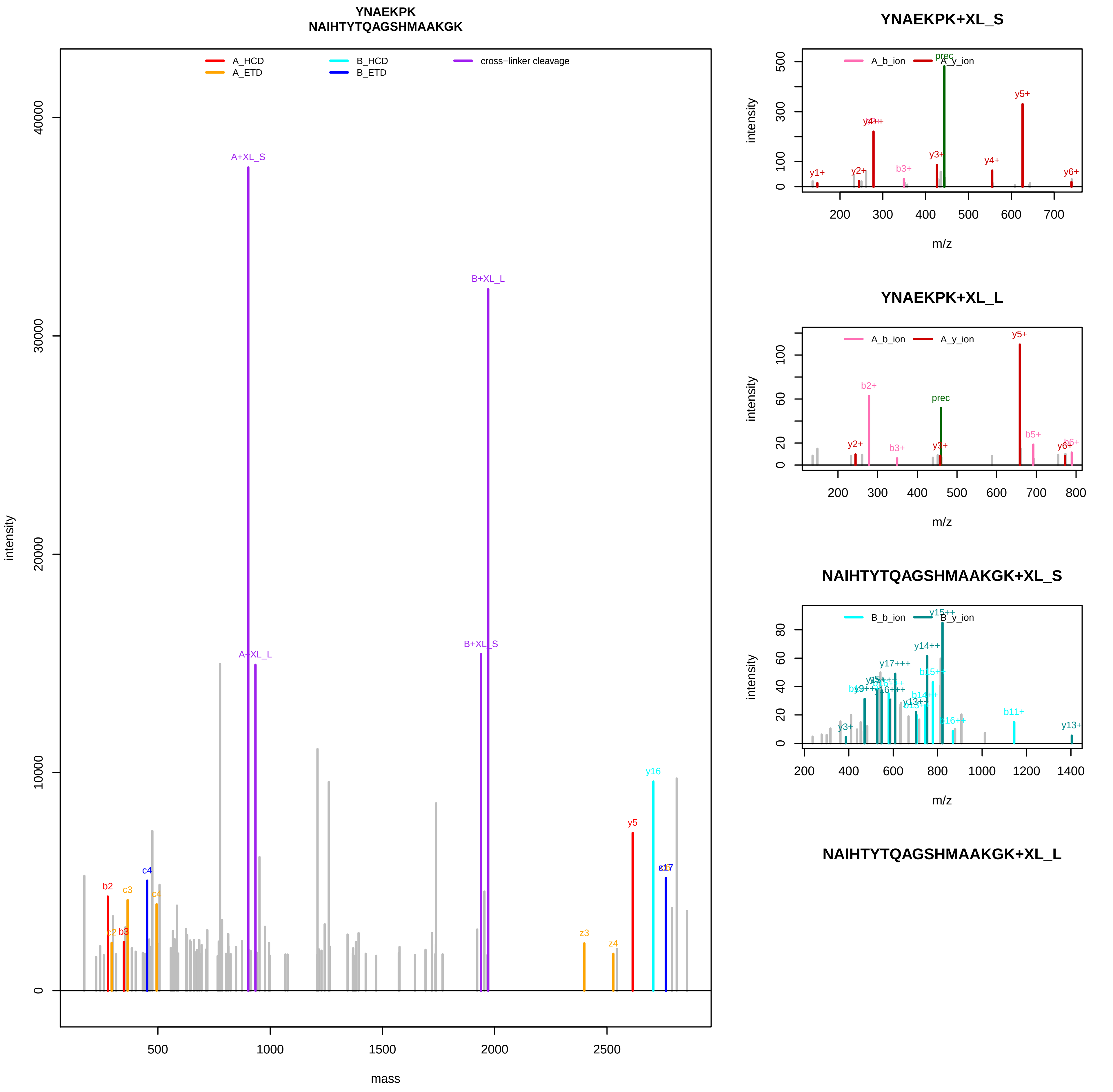
YNAEKPK
NAIHTYTQAGSHMAAKGK
A_HCD
A_ETD
B_HCD
B_ETD
cross−linker cleavage
A+XL_S
B+XL_L
A+XL_L
B+XL_S
b2
c2
b3
c3
c4
y16
y5
z3
z4
intensity
mass
0
10000
20000
30000
40000
500
1000
1500
2000
2500
YNAEKPK+XL_S
A_b_ion
A_y_ion
prec
y1+
y2+
b3+
y3+
y4+
y5+
y6+
m/z
YNAEKPK+XL_L
b2+
b5+
NAIHTYTQAGSHMAAKGK+XL_S
B_b_ion
B_y_ion
y15++
y14++
y17+++
b15++
b14++
y13++
b16++
b11+
y13+
y3+
NAIHTYTQAGSHMAAKGK+XL_L
